# Efficacy of moxibustion for pre- or stage I hypertension: study protocol for a pilot randomized controlled trial

**DOI:** 10.1186/1745-6215-13-188

**Published:** 2012-10-08

**Authors:** Kyung-Min Shin, Ji-Eun Park, Yan Liu, Hee-Jung Jung, So-Young Jung, Min-Hee Lee, Kyung-Won Kang, Tae-Han Yook, Sun-Mi Choi

**Affiliations:** 1 Medical Research Division, Korea Institute of Oriental Medicine, Daejeon, South Korea; 2 University of Science & Technology, Daejeon, South Korea; 3 Department of Acupuncture & Moxibustion, Woosuk University Hospital of Oriental Medicine, Jeonju, South Korea

**Keywords:** Moxibustion, Hypertension, Prehypertension

## Abstract

**Background:**

Hypertension is a risk factor for cardiovascular disease, and the prevalence of hypertension tends to increase with age. Current treatments for hypertension have adverse side effects and poor adherence. The purpose of this study is to evaluate the effects of moxibustion on blood pressure in individuals with pre- or stage I hypertension.

**Methods/design:**

Forty-five subjects with pre- or stage I hypertension will be randomized into three groups: treatment group A (2 times/week), treatment group B (3 times/week), and the control group (non-treated group). The inclusion criteria will be as follows: (1) aged between 19 and 65 years; (2) prehypertension or stage I hypertension (JNC 7, Seventh Report of the Joint National Committee on the Prevention, Detection, Evaluation, and Treatment of High Blood Pressure); (3) the participants are volunteers and written consent obtained.

The participants in the treatment group A will undergo indirect moxibustion 2 times per week for 4 weeks, and the participants in the treatment group B will undergo indirect moxibustion 3 times per week for 4 weeks. The participants in the control group (non-treated group) will maintain their current lifestyle, including diet and exercise. The use of antihypertensive medication is not permitted. The primary endpoint will be a change in patient blood pressure. The secondary endpoints will be the body mass index, lipid profile, EuroQol and Heart Rate Variability. The data will be analyzed with the Student’s t-test and analysis of variance (ANOVA) (*p* < 0.05).

**Discussion:**

The results of this study will help to establish the optimal approach for the care of adults with pre- or stage I hypertension.

**Trial registration:**

Clinical Research Information Service KCT0000469

## Background

Hypertension is an important risk factor for cardiovascular diseases including stroke, myocardial infarction, congestive heart failure, kidney disease, and peripheral vascular disease [1-3].

It is estimated that in 2025, 1.56 billion adults will have hypertension [4]. According to the Seventh Report of the Joint National Committee on the Prevention, Detection, Evaluation, and Treatment of High Blood Pressure(JNC-7), patients with a blood pressure of 120–139/80–89 mmHg have prehypertension [5]. A recent study demonstrated that people with prehypertension were more likely to develop hypertension than those with normal blood pressure over the 50-year follow-up period [6]. According to a longitudinal population-based US cohort study, prehypertension increases the risk of developing cardiovascular diseases [7]. The results of another study suggest that prehypertension increases the risk of myocardial infarction, stroke, cardioplegia, and cardiovascular death in women [8].

Moxibustion involves the application of ignited mugwort (*Artemisia vulgaris*) at acupoints or other specific parts of the body to treat or prevent diseases. This technique is typically used to treat and prevent cold syndrome, deficiency conditions and chronic diseases [9]. Moxibustion can cause tissue damage as a result of skin irritation or even skin burns due to the thermal stimulation, which is performed at various temperatures. The thermal stimulation can also cause inflammatory responses and vascular changes. According to the findings of previous studies of moxibustion, mediators such as histamine and substance P are secreted, and this process promotes angiectasis [10]. It seems that inflammatory reactions that affect vascular activity may ease various cardiovascular diseases, including hypertension [11].

There are few published randomized controlled trials (RCTs) concerning moxibustion treatment for prehypertension. A systematic review on the effects of moxibustion on hypertension revealed no evidence that moxibustion is beneficial to people with hypertension. However, the differences between specific and non-specific effects should be examined in a future study that includes appropriate control groups [11].

## Methods/design

### Aims of the study

The aims of this pilot study are to evaluate the effects of moxibustion on blood pressure in patients with pre- or stage I hypertension, to test the methods used and to calculate the sample size required for future randomized trials.

### Design

This study will be a prospective randomized controlled trial, and participants will be recruited from Woo-suk University Hospital using notices at the hospital and newspaper advertisements.

**Figure 1:**
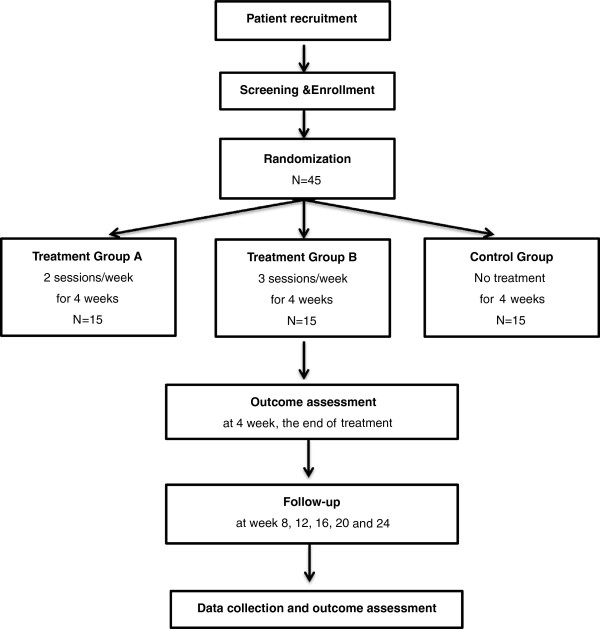
Flowchart of the study design.

In this pilot clinical trial, patients with pre- or stage I hypertension will be analyzed to determine the safety and efficacy of moxibustion, and basic analysis will be performed on the results. The study participants will voluntarily sign clinical trial consent forms, and all examinations and tests will be performed according to the clinical trial plan. Eligible subjects will be chosen based on specific inclusion and exclusion criteria. Participants will be randomized before the first treatment. Forty-five subjects with pre- or stage I hypertension will be randomized into three groups: treatment group A (2 times/week), treatment group B (3 times/week), and the control group (non-treated group). During each moxibustion treatment, each acupoint will be treated five times. The treatment will continue for 4 weeks. The follow-up assessment is designed to evaluate the long-term effects of moxibustion. Upon completion of the 4-week-long treatment, follow-up tests will be conducted at weeks 8,12,16,20 and 24 after randomization (Figure 1).

This study has been registered with the ‘Clinical Research Information Service,’ Republic of Korea, which is a registry in the WHO Registry Network (KCT0000469).

The systolic and diastolic blood pressures will be measured in the arm using an automatic blood pressure meter (HD-505, Jawon Medical Co, Kyungsan City, Korea). The subjects will be fitted with a BP cuff on both arms. A trained researcher will measure the blood pressure carefully in the arm with higher BP while the subject is in the seated position. The blood pressure will be measured three times after the subject has rested for at least 10 min. All BP measurements will be taken in a temperature-controlled room. The three measurements will be carried out at 5-min intervals. The mean of these three measurements will be used in the data analysis. The participants will be instructed not to drink caffeinated drinks such as coffee or tea and not to exercise, smoke or eat 2 h before the blood pressure test. In addition, they will be instructed to avoid drinking heavily the day before the test [12].

The practitioner has had more than 3 years of clinical experience since completing 6 years of study at the College of Korean Medicine and has received a Doctor of Korean Medicine.

### Randomization

This study is designed as a pilot study to calculate the appropriate sample size for future randomized clinical trials. Allowing for a 20% dropout rate, each group will include 15 participants, which is the minimum sample size necessary to evaluate the effect of moxibustion [13,14]. In this study, stratified randomization will be performed by classifying subjects into age and sex by an online centralized randomization service.

### Ethics

This protocol adheres to the principles of the Declaration of Helsinki and has been approved by the institutional review boards of the Woo-suk University Hospital, where the study will take place (WSOH IRB 1205-02). Before any treatment is given, written consent will be obtained from each participant. All patients will have the right to withdraw from the trial at any time.

### Participants

Inclusion criteria were as follows: (1) aged between 19 and 65 years; (2) pre- or stage I hypertension (JNC 7); (3) the participants are volunteers, and written consent was obtained.

Exclusion criteria were as follows: (1) taking medications to control blood pressure; (2) secondary hypertension; (3) history of cerebrovascular disease, cardiovascular disease, malignant tumors, kidney diseases, liver diseases, thyroid gland diseases, active tuberculosis, or other infectious diseases; (4) diabetic patients taking insulin or anti-diabetic medications; (5) drug or alcohol dependence; (6) taking hemorrhagic disease and anticoagulation medications (excluding aspirin); (7) receiving systemic steroid therapy or immunosuppressive therapy; (8) taking medications that could affect blood pressure such as central nervous system depressants or stimulants; (9) pregnancy or the possibility of pregnancy; (10) hypersensitivity reactions following moxibustion therapy; (11) received oriental medicine treatment related to hypertension in the past month; (12) participants deemed unsuitable for the trial, as judged by the person in charge of the clinical trial (Table 1).

**Table 1:** Patient inclusion and exclusion criteria

**Inclusion criteria**		**Exclusion criteria**	
1.	Aged between 19 and 65 years	1.	Taking medications to control blood pressure
2.	Pre- or stage 1 hypertension (JNC 7)	2.	Secondary hypertension
	-Systolic blood pressure between 120 and 159 mmHg	3.	History of cerebrovascular disease, cardiovascular disease, malignant tumors, kidney diseases, liver diseases, thyroid gland diseases, active tuberculosis, or other infectious diseases
	- Diastolic blood pressure between 80 and 99 mmHg	4.	Diabetic patients taking insulin or anti-diabetic medications
3.	The participants are volunteers and written consent obtained	5.	Drug or alcohol dependence
		6.	Taking hemorrhagic disease and anticoagulation medications (excluding aspirin)
		7.	Receiving systemic steroid therapy or immunosuppressive therapy
		8.	Taking medications that could affect blood pressure such as central nervous system depressants or stimulants
		9.	Pregnancy or the possibility of pregnancy
		10.	Hypersensitivity reactions following moxibustion therapy
		11.	Received oriental medicine treatment related to hypertension in the past month
		12.	Participants deemed unsuitable for the trial, as judged by the person in charge of the clinical trial.

### Interventions

#### Treatment group A

The subjects receiving moxibustion treatment will be treated 2 times per week.

During each moxibustion treatment, each acupoint will be treated five times. The moxibustion treatment will last for 25 min. The treatment will continue for 4 weeks. Following the procedures used in several previous studies [11,15,16], acupuncture needles will be inserted bilaterally into three acupuncture points (LI11, ST36, GB39) on the four peripheral extremities and unilaterally into two points (CV4, CV12) in the abdominal region. Indirect moxibustion (Manina, Haitnim Co., Ltd., Korea. 19 mm diameter and 21 mm height) will be used in the trial.

#### Treatment group B

The format will be exactly the same as those for treatment group A, but the subjects receiving moxibustion treatment will be treated three times per week.

### Control group (untreated group)

The control group (untreated group) will receive no treatment. The subject in this group will be asked to maintain their normal lifestyle, including diet, exercise and workload.

### Permitted and prohibited concomitant treatments

Brochures containing information on dietary changes, living habits, and exercise regimens that help prevent and alleviate hypertension will be distributed to all groups, and the subjects will decide for themselves whether to incorporate the suggested changes into their daily lives.

All groups will be prohibited from undergoing active treatment to lower the blood pressure for the duration of their participation in this clinical trial. After the completion of the clinical trial, during the assessment period, the subjects will be allowed to decide for themselves whether to receive additional treatment. Any other related information will be recorded in detail.

The use of birth control pills and central nervous system stimulants or depressants, which can affect blood pressure, will remain prohibited. The practitioner will be allowed to converse with the subjects about daily care and treatment details while carrying out the required examination.

### Outcome measures

The primary outcome measurement in this study is the change in blood pressure before randomization (baseline) and 4 weeks after randomization. Blood pressure will be taken at every visit.

The secondary outcome measures will be the mean pulse pressure, body mass index (BMI), heart rate variability (HRV), the Modified Stress Response Inventory (SRI-MF), the Pittsburgh Sleep Quality Index (PSQI), the Fatigue Severity Scale (FSS), EuroQol (EQ-5D), general assessments and blood tests including fasting blood sugar (FBS), uric acid, lipid profile, high sensitivity C-reactive protein (hs-CRP), liver function test (LFT), hemoglobin A1c (HbA1C), and complete blood count (CBC).

The pulse pressure refers to the difference between the systolic pressure and the diastolic pressure. This value reflects the degree of stiffness of the blood vessels. An increase in the pulse pressure after midlife is recognized as a risk factor for cardiovascular diseases [17]. The BMI will be calculated, and laboratory tests including FBS, uric acid, lipid profile, LFT, HbA1C, and CBC will be performed before and after treatment. The subjects will fast for 12 h before blood is drawn for these tests. hs-CRP is an index used to assess the degree of risk for cardiovascular diseases and to determine prognosis [18]. After 10 min of relaxation, the HRV will be measured with electrodes attached to both wrists and ankles using QECG-3 (LXC3203, Laxtha Inc., Korea) to obtain the LF/HF ratio, TP, VLF, LF, HF, heart rate, SDNN, RMSSD, HRV index, and PNN50. The neck disability index (NDI) consists of ten questions: seven about whether the subject is able to perform functional activities, two about symptoms, and one about concentration [19]. The EQ-5D is a questionnaire that is used to assess the quality of life [20]. The subjects mark the best answers for five questions on mobility, self-care, pain, usual activities, and psychological status. The answers are given in the form of numbers (1 = no problem, 2 = moderate problem, 3 = severe problem), and the sum of the five numbers reflects the subject’s health status. Global assessments of the patients will be used to assess improvement following the treatment, as judged by the subjects [21]. The subjects can choose from five answers for questions on the degree of improvement or worsening of the blood pressure: “improved a lot,” “somewhat improved,” “no change,” “somewhat worsened,” and “worsened a lot.” The SRI-MF consists of 22 questions that are related to somatization (9 items), depression (9 items), and anger (3 items) [22]. The subjects will mark the best answer for each of the questions based on their experience in the past week. The assessment is performed by adding up the scores of each item to obtain a total score. The PSQI was used to assess the quality of sleep and to identify sleep disorders [23]. The PSQI is an index used to assess the quality of sleep and the presence of any sleep disorders in the past month. This questionnaire assesses seven items: subjective quality of sleep, latency, sleep duration, habitual sleep efficiency, factors that disturb sleep, use of sleeping aids, and impediments to daytime functioning. The FSS is a questionnaire consisting of nine items that is used to investigate the severity of fatigue during the past week [24,25]. A Pattern Identification questionnaire was developed specifically to determine whether there are differences in the distributions of the change in symptoms among the groups (Table 2).

**Table 2:** Content of baseline and follow-up questionnaires

**Measures**	**Baseline**	**End of treatment**	**Follow-up**
		**at 4 weeks after randomization**	**at 8, 12, 16, 20 and 24 weeks after randomization**
Informed consent	x	-	-
Inclusion/exclusion criteria	x	-	-
Randomization	x	-	-
Demographic information taking	x	-	-
Blood pressure	x	x	x
Blood test	x	x	-
General assessment	-	x	-
BMI (body mass index)	x	x	x
NDI (neck disability index)	x	x	x
EQ-5D (EuroQol)	x	x	x
PSQI (Pittsburgh Sleep Quality Index)	x	x	x
HRV (heart rate variability)	x	x	x
Questionnaire of pattern identification	x	x	x
SRI-MF (modified form of the Stress Response Inventory)	x	x	x
FSS (Fatigue Severity Scale)	x	x	x
Adverse events	-	x	x

### Follow-up

Follow-up tests will be conducted at 8, 12, 16, 20 and 24 weeks after randomization. The follow-up assessment is designed to evaluate the long-term effects of moxibustion.

### Statistical analysis

The results of the intention-to-treat (ITT) analysis will be used to assess the validity of the study as a whole. The per-protocol (PP) analysis results will be used as a reference. The ITT analysis will be used as the main safety assessment technique.

Continuous data will be represented by the average, standard deviation, minimum value, and maximum value, whereas categorical data will be represented by a frequency table. For the comparison of the results among the groups, the analysis of variance (ANOVA) test will be used when the data are normally distributed; the Kruskal-Wallis test will be used otherwise. In addition, the chi-square test will be performed for categorical data.

After 4 weeks, the differences in systolic and diastolic pressure will be summarized for each group using descriptive statistics, including the median, average, standard deviation, and interquartile range. In addition, we will evaluate the effectiveness of the treatment using an analysis of covariance as the dependent variable, the baseline score as the covariate, the group as the fixed factor, and the hypertension stage as the stratified variable. In addition, we will compare the results for the stratified groups with the results for the whole subject pool (non-stratified) to determine whether hypertension stage is a suitable stratification variable. The differences in the systolic pressure and diastolic pressure following the treatment for each group will be analyzed using a paired t-test or a Wilcoxon signed rank test, and the 95% confidence interval will be presented. To assess the difference in the tendency for each visit, repeated measures analysis of variance will be performed. A significance level of 5% will be used in all analyses.

The average pulse pressure, change in the baseline blood pressure following the treatment, BMI, EQ-5D, Ankle Brachial Pressure Index(ABI index), Brachial-Ankle Pulse wave velocity(PWV index), heart rate variation (LF/HF ratio, TP, VLF, LF, HF, heart rate, SDNN, LF norm, HF norm), and blood test results will be analyzed using the same techniques used to analyze the validity of the assessment variable. To determine whether there are differences in the distribution of the change in symptoms among the groups, analysis will be performed on each item using the chi-squared test.

All adverse reactions manifested will be listed with detailed explanations. The frequency of abnormal reactions that are correlated with the treatment and abnormal reactions that do not have such correlations will be recorded. A Fisher’s exact test will be performed to determine whether there are any differences among the groups with respect to the incidence of abnormal reactions as reported by the subjects. Furthermore, technical analysis will be performed to identify differences in the degree of severity and in the type of abnormal reactions among the groups.

### Adverse events

The safety evaluation will be based on adverse events, which are expected to include blisters, redness, and burns. Adverse reactions refer to undesirable and unintentional signs (e.g., abnormal test results), symptoms or diseases following the treatment. There does not necessarily have to be a cause and effect relationship with the treatment. The subjects will be instructed to voluntarily report any information regarding abnormal reactions to the practitioner on a regular basis.

Any adverse events during treatment will be recorded. When abnormal reactions appear, the date of appearance, the date of disappearance, the degree of the abnormal reaction, measures taken related to the treatment, correlation with the treatment, names of suspicious drugs taken outside of the treatment, whether the abnormal reactions were treated and any other related information will be recorded in detail. Any medical conditions or diseases present prior to the start of the treatment will be considered abnormal reactions only if they worsen after starting the treatment.

Abnormal test values or results will be considered abnormal reactions only if they cause clinical symptoms, if they are considered clinically significant or if treatment is needed.

## Discussion

This study is a preliminary study targeting individuals with pre- or stage I hypertension, who will be randomly divided into treatment group A (2 times/week), treatment group B (3 times/week), and the control group (non-treated group). Basic analysis will be performed to assess the validity and safety of moxibustion for the treatment of pre- or stage I hypertension. The following results will be presented: the extent of stimulation provided by moxibustion, differences in the effect according to the frequency of treatment, and how long the efficacy of the treatment last. These results will be based on long-term follow-up observation.

### Trial status

This trial is currently recruiting participants.

## Abbreviations

JNC-7: Seventh Report of the Joint National Committee on the Prevention, Detection, Evaluation, and Treatment of High Blood Pressure; RCTs: Randomized controlled trials; BMI: body mass index; HRV: heart rate variability; SRI-MF: modified stress response inventory; PSQI: Pittsburgh Sleep Quality Index; FSS: Fatigue Severity Scale; EQ-5D: EuroQol; FBS: fasting blood sugar; LFT: liver function test; hs-CRP: high sensitivity C-reactive protein; HbA1C: hemoglobin A1c; CBC: complete blood count; NDI: neck disability index; ITT: intent to treat; PP: per protocol; ANOVA: analysis of variance; ABI index: ankle brachial pressure index; PWV index: brachial-ankle pulse wave velocity.

## Competing interests

The authors declare that they have no competing interests.

## Authors’ contributions

KMS participated in the design of the study, coordinates the study and drafted the manuscript. JEP, YL, HJJ, SYJ, MHL, KWK, THY, and SMC provided technical advice and wrote the relevant sections of the manuscript. All authors participated in read, and approved the final manuscript.

## Authors’ information

KMS, JEP and KWK: Senior researcher of Medical Research Division, Korea Institute of Oriental Medicine.

SMC: Director of Medical Research Division, Korea Institute of Oriental Medicine.

YL, HJJ, SYJ and MHL: Researcher of Medical Research Division, Korea Institute of Oriental Medicine.

THY: Professor of the Department of Acupuncture & Moxibustion, Woosuk University.
